# Intrauterine ultrasound phenotyping, molecular characteristics, and postnatal follow-up of fetuses with the 15q11.2 BP1-BP2 microdeletion syndrome: a single-center, retrospective clinical study

**DOI:** 10.1186/s12884-023-06223-y

**Published:** 2024-01-03

**Authors:** Meiying Cai, Aixiang Lv, Wantong Zhao, Liangpu Xu, Na Lin, Hailong Huang

**Affiliations:** Medical Genetic Diagnosis and Therapy Center, Fujian Maternity and Child Health Hospital College of Clinical Medicine for Obstetrics & Gynecology and Pediatrics, Fujian Medical University, Fujian Key Laboratory for Prenatal Diagnosis and Birth Defect, Fuzhou, China

**Keywords:** 15q11.2 BP1-BP2 microdeletion syndrome, Prenatal phenotyping, Postnatal follow-up, Mental retardation, Copy number variation

## Abstract

**Objectives:**

The 15q11.2 BP1-BP2 microdeletion is associated with neurodevelopmental diseases. However, most studies on this microdeletion have focused on adults and children. Thus, in this study, we summarized the molecular characteristics of fetuses with the 15q11.2 BP1-BP2 microdeletion and their postnatal follow-up to guide prenatal diagnosis.

**Methods:**

Ten thousand fetuses were retrospectively subjected to karyotype analysis and chromosome microarray analysis.

**Results:**

Chromosome microarray analysis revealed that 37 (0.4%) of the 10,000 fetuses had 15q11.2 BP1-BP2 microdeletions. The fragment size of the 15q11.2 BP1-BP2 region was approximately 312–855 kb and encompassed *TUBGCP5*, *CYFIP1*, *NIPA2*, and *NIPA1* genes. Twenty-five of the 37 fetuses with this microdeletion showed phenotypic abnormalities. The most common ultrasonic structural abnormality was congenital heart disease, followed by renal dysplasia and Dandy–Walker malformation. The 15q11.2 BP1-BP2 microdeletion was inherited from the father and mother in 6 and 10 cases, respectively, and de novo inherited in 4 cases. In the postnatal follow-up, 16.1% of the children had postnatal abnormalities.

**Conclusion:**

Fetuses with the 15q11.2 BP1-BP2 microdeletion showed high proportions of phenotypic abnormalities, but the specificity of penetrance was low. Thus, fetuses with this syndrome are potentially at a higher risk of postnatal growth/behavioral problems and require continuous monitoring of growth and development.

## Background

The 15q11.2 BP1-BP2 microdeletion is the most common pathogenic copy number variation (CNV) associated with neurodevelopmental diseases, cognition, behavior, and brain structure and morphology changes [1, 2]. In 2004, Bulter et al. [3] identified 15q11.2 BP1-BP2 as a region susceptible to neuropathic phenotypes; since then, the relationship between copy number changes in this segment and diseases has gained significant attention. Two patients with this segment deletion belonged to one family and it was suggested that this segment deletion might be pathogenic [4]. Statistically significant differences in the detection rate of this segment between patients with multiple disease spectra and healthy population were observed [4–6]. In 2015, a study summarized the main clinical phenotypes of 200 children with the 15q11.2 BP1-BP2 microdeletion syndrome: (1) developmental delay and language delay; (2) ear deformity and cleft palate; (3) writing and reading difficulties, memory problems and verbal IQ scores ≤ 75; (4) general behavior problem or undetermined; and (5) abnormal brain structure; other clinical features include epilepsy, autism spectrum disorder (ASD), schizophrenia/paranoid psychosis, and motor retardation [7]. In summary, the phenotypes of the 15q11.2 BP1-BP2 microdeletion syndrome are diverse with clear individual differences.

The phenotypes of the 15q11.2 BP1-BP2 microdeletion syndrome are varied and not completely explicit; therefore, it can be inherited from parents with a normal phenotype. The pathogenicity of this syndrome is unclear and some studies report it as a CNV with unknown clinical significance [8, 9]. However, cohort studies show that the 15q11.2 BP1-BP2 microdeletion is the most common genetic cause of ASD and schizophrenia [2, 10]; 15q11.2 BP1-BP2 is a susceptible region site for schizophrenia [11, 12] and developmental delay [13], and is associated with epilepsy [14, 15]. Over 80% of 15q11.2 BP1-BP2 microdeletion carriers show developmental delays [16, 17]. The ClinGen database has rated it as a pathogenic CNV in 2021 [18]. The widespread application of chromosome microarray analysis (CMA) in prenatal diagnosis has identified an increasing number of pathogenic CNVs, giving a diagnostic yield up to 20%, with 15q11.2 BP1-BP2 having the highest percentage of microdeletions [19]. However, these studies were performed on adults and children; fetuses have not been studied in this regard.

Therefore, in the present study, we reviewed and analyzed the intrauterine ultrasound phenotypes of fetuses with the 15q11.2 BP1-BP2 microdeletion syndrome and their postnatal follow-up to provide a basis for prenatal diagnosis and postnatal monitoring of fetuses with this syndrome in the future.

## Methods

### Patient recruitment

This was a retrospective clinical study. From November 2016 to December 2022, 10,000 fetuses had undergone invasive prenatal diagnosis at the Prenatal Diagnosis Center of Fujian Maternal and Child Health Hospital for traditional karyotype analysis and CMA. All pregnant women included in the study met the criteria for prenatal invasive diagnostic procedures; after the purpose of invasive diagnosis was clarified to the pregnant women and their families, the women accepted to undergo the examination and signed the informed consent form. Patients with prenatal data defects and patients with clinical data defects were excluded. Indication of prenatal diagnosis included advanced age of maternal age, fetal ultrasound abnormality, abnormal in serological screening, abnormal in noninvasive prenatal screening, and histories of abnormal pregnancy. We confirmed the presence of 15q11.2 BP1-BP2 microdeletions in 37 fetuses using CMA. The pregnant women were aged from 19 to 45 years, and the gestational period ranged from 18 to 34 weeks. Samples from the pregnant women for prenatal diagnosis were collected through the abdominal wall under ultrasound guidance during different gestational weeks. Amniocentesis was performed at 18–24 weeks of pregnancy, and umbilical cord blood sampling was performed after 24 weeks of pregnancy. All pregnant women received genetic counseling before testing and signed informed consent forms. This study was approved by the Medical Ethics Committee of Fujian Maternal and Child Health Hospital (no. 2014042). All pregnant women received genetic counseling after testing.

### Karyotype analysis

The extracted amniotic fluid was divided among two aseptic centrifuge tubes and centrifuged at 2,000 rpm for 10 min. Subsequently, the supernatant was discarded and mixed with 4 mL of medium. The fluid was inoculated in two culture bottles and incubated at 37°C in a 5% CO_2_ incubator. Amniotic fluid cells were harvested after 8–10 days of culture. The cells were digested with pancreatic enzymes and transferred to a centrifuge tube. The sample was centrifuged at 2,000 rpm and 10 min of hypoosmosis with 0.075 mol/L potassium chloride (1:1); 1 mL of fixative was added for prefixation. The sample was centrifuged at 2,000 rpm for 10 min at 37 °C, the supernatant was discarded and fixed twice, and conventional preparation and G-banding were performed. Umbilical cord blood was inoculated and directly cultured in an incubat or at 37 °C. The cells were harvested after 3 days in the same manner as the amniotic fluid cells. Karyotype analysis was conducted according to the International System for Human Cytogenetics Nomenclature. Twenty cell phases were counted, and five karyotypes were analyzed. Chimeras increased the count.

### CMA

Fetal genomic DNA was extracted according to the manufacturer’s instructions. Enzyme digestion, linking, amplification, purification, fragmentation, labeling, hybridization, and scanning of the fetal genomic DNA were performed in strict accordance with the standard operating procedures of an Affymetrix CytoScan 750 K array chip. Data obtained by scanning were analyzed using the Chromosome Analysis Suite software. The databases of International Standards for Cytogenomic Arrays, National Center for Biotechnology Information, Database of Chromosomal Imbalance and Phenotype in Humans Using Ensembl Resources, and Online Mendelian Inheritance in Man (OMIM), and other public databases were compared and analyzed to determine the nature of the detected CNV. Based on the results, the CNVs were divided into pathogenic CNV, likely pathogenic CNV, uncertain clinical significance CNV, likely benign CNV, and benign CNV. Parents of fetuses with abnormal CNV were advised to get peripheral blood CMA tests done to determine whether the fetal CNV was inherited from parents or was *denovo*.

### Follow-up

We followed up with the 37 cases with the 15q11.2 BP1-BP2 microdeletion syndrome to record the gestational state of the pregnant mothers and the postnatal state of the fetuses. All fetuses were followed up after birth, mainly for growth deviation and developmental delay.

## Results

### CMA and parental verification

**Fig. 1:**
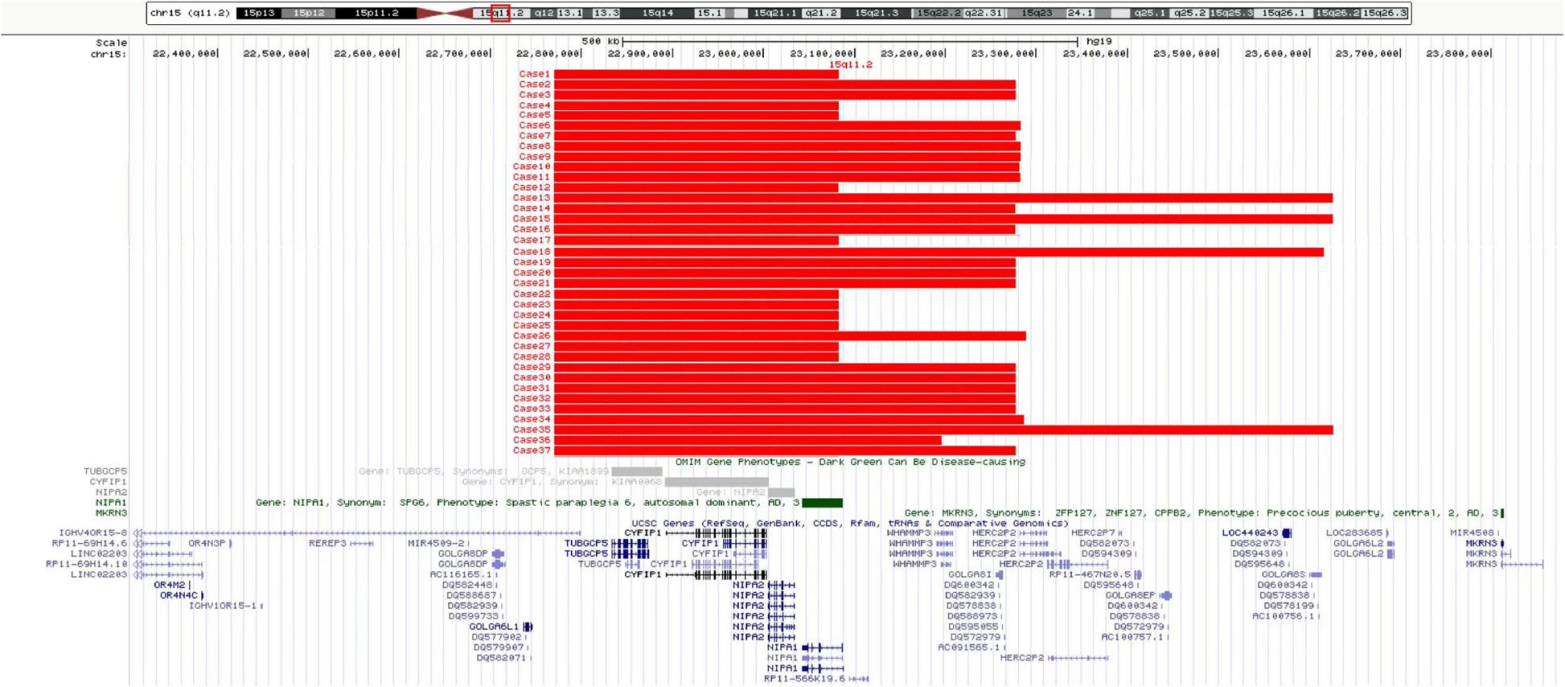
Chromosome microarray analysis (CMA) results revealed that 37 fetuses had a 15q11.2 BP1-BP2 microdeletion, with a fragment size of 312–855 kb, containing four Online Mendelian Inheritance in Man (OMIM) genes: *TUBGCP5*, *CYFIP1*, *NIPA2*, and *NIPA1*

CMA revealed that 37 (0.4%) of the 10,000 fetuses had a 15q11.2 BP1-BP2 microdeletion, with a fragment size of 312–855 kb, containing four OMIM genes: *TUBGCP5*, *CYFIP1*, *NIPA2*, and *NIPA1* (Fig. 1). Among these 37 fetuses, 23 were male (62.2%,) and 14 were female (37.8%). After genetic counseling, the parents of 17 cases refused pedigree verification. Parental verification of the parents of the remaining 20 fetuses revealed that 15q11.2 BP1-BP2 microdeletions were inherited from normal phenotype mothers in 10 cases, normal phenotype fathers in 6 cases, and *denovo* in 4 cases. Thus, the male-to-female ratio of the parents that transferred this microdeletion to fetuses was 3:5. We divided the clinical characteristics and follow-up results of the 37 fetuses according to ultrasonic phenotype with normal ultrasonic phenotype (Table 1), ultrasonic soft markers (Table 2), and abnormal ultrasonic structure (Table 3).

**Table 1:** Clinical characteristics and follow-up results of fetuses with normal ultrasonic phenotype

Case	Indication of prenatal diagnosis	Karyotype analysis	CMA	Size(Kb)	Inheritance	Follow-up
1	Advanced age	46,XX	arr[hg19]15q11.2(22,770,421–23,082,237) × 1	312	Refused	1 year and 4 months, Normal
2	Advanced age	46,XY	arr[hg19]15q11.2(22,770,421–23,277,436) × 1	507	Maternal	1 year and 3 months, Normal
3	Advanced age	46,XX	arr[hg19]15q11.2(22,770,421–23,277,436) × 1	507	Paternal	1 year, Normal
4	Advanced age	46,XY	arr[hg19]15q11.2(22,770,422–23,082,237) × 1	312	Refused	1 year and 4 months, Normal
5	Advanced age	46,XY	arr[hg19]15q11.2(22,770,422–23,082,237) × 1	312	*denovo*	2 months, Normal
6	Advanced age	46,XX	arr[hg19]15q11.2(22,770,421–23,282,798) × 1	512	*denovo*	2 years, Normal
7	High risk for Down's syndrome	46,XY	arr[hg19]15q11.2(22,770,421–23,625,785) × 1	855	Refused	3 years, Normal
8	High risk for Down's syndrome	46,XY	arr[hg19]15q11.2(22,770,421–23,277,436) × 1	507	*denovo*	Termination of pregnancy
9	High risk for Down's syndrome	46,XY	arr[hg19]15q11.2(22,770,421–23,625,785) × 1	855	Refused	3 years, Normal
10	High risk for Down's syndrome	46,XY	arr[hg19]15q11.2(22,770,421–23,276,833) × 1	506	Refused	Termination of pregnancy
11	Poor fertility history	46,XX	arr[hg19]15q11.2(22,770,422–23,082,237) × 1	312	Refused	Termination of pregnancy
12	Family history of hemophilia	46,XY	arr[hg19]15q11.2(22,770,421–23,615,769) × 1	845	Refused	Termination of pregnancy

**Table 2:** Clinical characteristics and follow-up results of fetuses with ultrasonic soft markers

Case	Ultrasonic phenotype	Karyotype analysis	CMA	Size(Kb)	Inheritance	Follow-up
1	Thickened nuchal translucency	46,XX	arr[hg19]15q11.2(22,770,421–23,277,436) × 1	507	Maternal	1 year and 2 months, Short stature
2	Thickened nuchal translucency	46,XY	arr[hg19]15q11.2(22,770,421–23,282,798) × 1	512	Refused	1 year, Normal
3	Thickened nuchal translucency	46,XY	arr[hg19] 15q11.2(22,770,422–23,282,798) × 1	512	Refused	8 months, Normal
4	Thickened nuchal translucency	46,XY	arr[hg19] 15q11.2(22,770,421–23,281,886) × 1	511	Refused	3 years, Normal
5	Thickened nuchal translucency	46,XY	arr[hg19] 15q11.2(22,770,421–23,282,798) × 1	512	Refused	1 year and 3 months, Normal
6	Thickened nuchal translucency	46,XY	arr[hg19]15q11.2(22,770,422–23,082,237) × 1	312	Maternal	1 year and 6 months, Normal
7	Tricuspid regurgitation	46,XX	arr[hg19]15q11.2(22,770,421–23,277,436) × 1	507	Maternal	1 year and 4 months, Short stature
8	Tricuspid regurgitation	46,XY	arr[hg19]15q11.2(22,770,421–23,277,436) × 1	507	Maternal	6 months, Normal
9	Choroid plexus cyst	46,XY	arr[hg19]15q11.2(22,770,422–23,082,237) × 1	312	Maternal	5 months, Normal
10	Small for gestation age	46,XY	arr[hg19]15q11.2(22,770,422–23,082,237) × 1	312	Maternal	1 year, Normal
11	Thickened nuchal translucency	46,XY	arr[hg19]15q11.2(22,770,421–23,082,237) × 1	312	Refused	Termination of pregnancy
12	Absence of nasal bone	46,XX	arr[hg19]15q11.2(22,770,421–23,276,833) × 1	506	Paternal	4 years, Normal
13	Tricuspid regurgitation	46,XY	arr[hg19]15q11.2(22,770,421–23,277,436) × 1	507	Refused	4 years, Normal
14	Ventriculomegaly	46,XY	arr[hg19]15q11.2(22,770,421–23,277,436) × 1	507	Maternal	3 years, Normal
15	Tricuspid regurgitation	46,XX	arr[hg19]15q11.2(22,770,421–23,286,423) × 1	516	Paternal	4 years, Normal
16	Choroid plexus cyst, Ventriculomegaly	46,XY	arr[hg19]15q11.2(22,770,421–23,625,785) × 1	855	Paternal	4 years,language retardation
17	Thickened nuchal translucency	46,XX	arr[hg19]15q11.2(22,770,421–23,195,725) × 1	425	Maternal	2 years, Normal
18	Tricuspid regurgitation	46,XX	arr[hg19]15q11.2(22,770,421–23,277,436) × 1	507	Refused	2 years,language retardation,anal stenosis, underwent anoplasty

**Table 3:** Clinical characteristics and follow-up results of fetuses with abnormal ultrasonic structure

Case	Ultrasonic phenotype	Karyotype analysis	CMA	Size(Kb)	Inheritance	Follow-up
1	Congenital heart disease	46,XX	arr[hg19]15q11.2(22,770,421–23,277,436) × 1	507	Maternal	1 year and 2 months, Normal
2	Congenital heart disease	46,XY	arr[hg19]15q11.2(22,770,422–23,082,237) × 1	312	Refused	5 months, Normal
3	Congenital heart disease	46,XY	arr[hg19]15q11.2(22,770,422–23,082,237) × 1	312	*denovo*	5 months, Normal
4	Congenital heart disease	46,XY	arr[hg19]15q11.2(22,770,422–23,288,350) × 1	518	Paternal	1 year, Normal
5	Subcutaneous cystic mass at the posterior neck	46,XX	arr[hg19]15q11.2(22,770,421–23,082,237) × 1	312	Refused	4 years, Intellectual retardation,Rehabilitation therapy
6	Renal dysplasia	46,XX	arr[hg19]15q11.2(22,770,421–23,277,436) × 1	855	Refused	3 years, Normal
7	Dandy-Walker deformity	46,XX	arr[hg19]15q11.2(22,770,421–23,277,436) × 1	507	Paternal	Termination of pregnancy

### Karyotype analysis

No chromosomal abnormalities were detected in the karyotype analysis of the 37 fetuses with the 15q11.2 BP1-BP2 microdeletion.

### Intrauterine ultrasound phenotypes of fetuses with the 15q11.2 BP1-BP2 microdeletion syndrome

Among the 37 fetuses with the 15q11.2 BP1-BP2 microdeletion syndrome, 25 (67.6%) exhibited an abnormal phenotype in intrauterine ultrasound screening. The most common ultrasonic structural abnormality was congenital heart disease (five cases), followed by renal dysplasia and Dandy-Walker malformation (detected in one case each). The most common abnormal ultrasonic soft marker was thickened nuchal translucency, found in eight cases, followed by tricuspid regurgitation, observed in five cases. The remaining 12 fetuses with the 15q11.2 BP1-BP2 microdeletion syndrome showed no abnormal phenotype in intrauterine ultrasound screening, and the indications for prenatal diagnosis in these cases were advanced age in six cases, high risk for Down syndrome in four cases, poor fertility history in one case, and family history of hemophilia in one case.

### Obstetric and neonatal follow-up results

Among the parents of the 37 fetuses with the 15q11.2 BP1-BP2 microdeletion syndrome, the parents of 6 fetuses decided to terminate the pregnancy, while the parents of 31 fetuses continued the pregnancy after receiving adequate genetic counseling regarding the possible risks. We followed up these 31 fetuses after birth; the ages of these children ranged from 5 months to 4 years during the follow-up. Five of these children (16.1%) had postnatal abnormalities, including short stature (two), language retardation (one), intellectual retardation (one), and anal stenosis, in addition to language retardation (one); anal stenosis was revealed by physical examination at 6 months of age and the patient had undergone anoplasty. No abnormalities were detected in the remaining 26 cases (83.9%).

## Discussion

Chromosome 15 has five common breakpoints near the end of the long arm, often referred to as BP1-BP5. The breakpoints in Parder–William/Angelman syndrome are 15q11-q13 BP3 and BP1 or BP2, whereas the 15q11.2 BP1-BP2 microdeletion syndrome is in the centromeric region of the long arm of chromosome 15. It is adjacent to the Parder–William/Angelman syndrome imprinting area, and its size is approximately 500 kb, involving four OMIM genes: *NIPA1*, *NIPA2*, *CYFIP1*, and *TUBGCP5*, which are hot spots in clinical research [20–23]. *NIPA1* is highly expressed in neuronal tissues, and its variation leads to abnormal protein transport associated with ASD [24]. The *NIPA2* gene encoded protein plays an important role in the transport of Mg^2+^ in the kidneys and is overexpressed in B lymphocytes and the placenta, which is associated with Parder–William syndrome, ASD, and epilepsy [25]. The *TUBGCP5* gene is highly expressed in the heart and skeletal muscles and moderately expressed in the brain (mainly in the subthalamic nucleus) and is mostly related to Parder–Willi syndrome, followed by spermatogenesis and ASD; it is also associated with stress disorders [26]. *CYFIP1* is highly expressed in the perinuclear region and synaptosomes, is part of the ribonucleoprotein complex, and is associated with Fragile X syndrome and ASD [1]. Thus, language and/or motor delays are associated with dyslexia, mental/behavioral problems (such as ASD and schizophrenia), ataxia or poor coordination, epilepsy, congenital brain development abnormalities, and structural defects occur in 15q11.2 BP1-BP2 microdeletions. However, studies on these genes have failed to completely explain the phenotypes and penetrance of the 15q11.2 BP1-BP2 microdeletion syndrome. In this study, 15q11.2 BP1-BP2 microdeletion was found in 37 fetuses during the prenatal diagnosis of 9,000 fetuses. The fragment size was approximately 312–855 kb, and this region included the four genes mentioned above (*NIPA1*, *NIPA2*, *CYFIP1*, and *TUBGCP5*).

Among the 37 fetuses with the 15q11.2 BP1-BP2 microdeletion syndrome, 25 showed phenotypic abnormalities on intrauterine ultrasound (67.6%), among which the most common ultrasonic structural abnormality was congenital heart disease (five cases; 13.5%), followed by renal dysplasia and Dandy-Walker malformation in one case each. Fetal echocardiography was used to detect congenital heart disease [27]. Recently, 15q11.2 BP1-BP2 microdeletions and congenital heart disease were confirmed to be correlated; approximately 9% of 15q11.2 microdeletion syndrome cases are associated with different types of congenital heart disease [28–30]. In addition to the above ultrasound structural abnormalities, eight fetuses with 15q11.2 microdeletion syndrome showed a prenatal ultrasound phenotype of thickened nuchal translucency. During the fetal period, 5.6% of pathogenic CNVs exhibit thickened nuchal translucency [19]. However, further investigation is required to determine whether thickened nuchal translucency is related to the phenotype of fetuses with the 15q11.2 BP1-BP2 microdeletion syndrome. Additionally, this study found that the ultrasound phenotype of five fetuses with the 15q11.2 BP1-BP2 microdeletion syndrome was tricuspid valve regurgitation. No relevant studies have reported on 15q11.2 microdeletion and tricuspid valve regurgitation, and the relationship between the two remains unclear. The reported fetal ultrasound phenotypes correlate with the BP1-BP2 microdeletion syndrome, but the degree of correlation remains unknown, making prenatal diagnosis significantly challenging. Therefore, more data are needed to enrich the fetal stage phenotype spectrum of patients with 15q11.2 BP1-BP2 microdeletion syndrome and ultimately improve the sensitivity of prenatal diagnosis.

Most CNV are inherited from phenotypic parents, particularly from the paternal line [2, 7, 31]. This may be related to a female protective effect, wherein females at a genetic risk of exhibiting a neurodevelopmental disorder phenotype require a higher mutation load than males [22, 32]. In the present study, the 15q11.2 BP1-BP2 microdeletion syndrome in 16 fetuses was inherited from a phenotypically normal paternal or maternal line, 4 fetuses developed the syndrome de novo, and the parents of 17 fetuses refused parental verification. The 15q11.2 BP1-BP2 microdeletion was inherited from a normal phenotype mother in 10 cases and from a normal phenotypic father in 6 cases. The male-to-female ratio of the parents that transferred this syndrome was 3:5, which is not consistent with the findings of the existing literature. This deviation in the gender ratio in this study may be owing to the exclusion of 17 cases from parental verification. Further, 31 cases were followed up after birth, with the ages of the children ranging from 5 months to 4 years during the follow-up. Among them, five cases (16.1%) had postnatal abnormalities, including short stature (two cases), language retardation (one case), intellectual retardation (one case), and anal stenosis, along with language retardation (one case). No abnormalities were detected in the remaining 26 patients (83.9%). The results of this study were significantly different from those of previous reports, which may be owing to the small number of cases in this study and the lack of phenotypes in children owing to a short follow-up time. ASD and behavioral disorders in children are generally believed to become stable after 2 years of age [33] and the value of autism screening and language delay assessment in asymptomatic children under 5 years of age has been questioned [34]. The main phenotype of the 15q11.2 BP1-BP2 microdeletion syndrome reported in this study was developmental retardation occurring during postnatal growth and development, which requires long-term follow-up and evaluation.

This study had some limitations. First, the number of cases in this study was small, which may have caused bias. In the future, a higher number of cases should be collected for microdeletion-related studies. Second, the prognosis of children with the 15q11.2 BP1-BP2 microdeletion syndrome depends on neurodevelopmental, cognitive, and behavioral problems, and their age, severity, duration, and family genetic background. Therefore, regular, and long-term evaluations of developmental, language, and behavioral abilities are needed. We plan to regularly follow-up with the 31 children with the 15q11.2 BP1-BP2 microdeletion syndrome to achieve early detection and intervention of diseases and improve the long-term prognosis of these children.

## Conclusion

Phenotypic abnormalities in fetuses with the 15q11.2 BP1-BP2 microdeletion syndrome is high, and the most common abnormalities are congenital heart disease and thickened nuchal translucency; however, the specificity of penetrance remains low. The 15q11.2 BP1-BP2 microdeletion syndrome corresponds to a relatively broad spectrum of diseases, and it is difficult to identify fetal gene-phenotype associations using only existing prenatal diagnostic techniques. Fetuses with the 15q11.2 BP1-BP2 microdeletion syndrome are potentially at a higher risk of postnatal growth and development/behavioral problems and require continuous growth and development monitoring.

## Data Availability

All data generated during and/or analyzed during the current studyare available upon request by contacting the corresponding author.

## Abbreviations

ASD: Autism spectrum disorder
CMA: Chromosome microarray analysis
CNV: Copy number variation
OMIM: Online Mendelian Inheritance in Man
